# Relations Between Parental and Child Separation Anxiety: The Role of Dependency-Oriented Psychological Control

**DOI:** 10.1007/s10826-015-0122-x

**Published:** 2015-01-18

**Authors:** Lisanne L. Stone, Roy Otten, Bart Soenens, Rutger C. M. E. Engels, Jan M. A. M. Janssens

**Affiliations:** 1 Behavioural Science Institute, Radboud University Nijmegen, P.O. Box 9104, 6500 HE Nijmegen, The Netherlands; 2 Department of Developmental, Social, and Personality Psychology, Ghent University, Ghent, Belgium

**Keywords:** Separation anxiety, Psychologically controlling parenting, Child self-report, Childhood anxiety

## Abstract

Although separation anxiety is prevalent in young children, it remains unclear whether and how maternal separation anxiety is related to separation anxiety in children. This study examined associations between maternal separation anxiety and separation anxiety in children, and the potential effect psychologically controlling parenting. Mothers (N = 269) and children (N = 287) recruited for a community sample participated in two 1-year interval data-waves. Children were aged five-eight and were interviewed using an age-appropriate method for obtaining self-reports of separation anxiety and perceptions of dependency-oriented psychologically controlling parenting. Mothers reported on their feelings of separation anxiety regarding their child via a questionnaire. We found that maternal separation anxiety was positively related to separation anxiety in children within, but not over time. We did not find psychologically controlling parenting to mediate this association. Studying other factors than parenting may be an important avenue for future research in explaining separation anxiety in children.

## Introduction

Separation anxiety is a developmentally appropriate reaction of distress to separation of the caregiver during infancy and central to the child’s psychological development (Blatt 2004; Bowlby 1988; Mahler 2000). Although most children adequately learn to regulate their distress reaction to separation, some children continue to experience anxiety following separation. When symptoms of separation anxiety persist, these behaviors can become highly problematic and debilitating (Jurbergs and Ledley 2005). In this case, normative fears and worries concerning separation of the caregiver become non-age-appropriate and are associated to school refusal and excessive truancy (Egger et al. 2003; Kearney and Albano 2004). Separation anxiety disorder is the most common anxiety classification below the age of 12 (Cartwright-Hatton et al. 2006). It is an antecedent of adult anxiety disorders and linked to depression in young adults (Hirshfeld-Becker et al. 2008). Symptoms of separation anxiety have been found to be more influenced by the shared environment than by heritability; therefore, gaining insight into familial factors that may maintain or exacerbate separation anxiety seems to be very important (Eley et al. 2008).

In developmental theories, great importance is attributed to issues of separation between mother and child, as it is central to the development of the child’s psychological self in a process termed separation-individuation (Mahler 2000). Also, studying separation may be central to understanding aspects of parenting (Hock et al. 1989). Object relations theory proposes that there is an optimal maternal distance, suited to the infant’s changing developing needs (Blatt 2004; Mahler 2000). It is suggested that when mothers do not have a healthy sense of self, it is more difficult to see herself as separate from the child, and experiences of separation bring about personal feelings of loss or rejection (Hock and Schirtzinger 1992). This process is termed maternal separation anxiety (Hock et al. 1989). The threatening feelings regarding separateness that are characteristic of maternal separation anxiety may lead to more protective behaviors in the mother (Barber and Harmon 2002), and as such, impede the separation-individuation process in children (Blatt 2004). Although research has begun to study some of these theoretical propositions, the overall line of reasoning requires empirical testing. A few studies have investigated the link between maternal separation anxiety and separation anxiety in their children (Dallaire and Weinraub 2005; Hock et al. 2004; Mayseless and Scher 2000; Peleg et al. 2006). In a cross-sectional study, maternal perception of separation effects on her child was related to observed child separation anxiety in 38 preschoolers (Peleg et al. 2006). Further, maternal separation anxiety predicted symptoms of separation anxiety in 99 children at age six (Dallaire and Weinraub 2005), and prior maternal worry predicted feelings of anxiety in 48 11-year-olds (Hock et al. 2004). In contrast, maternal separation anxiety was not related to fearful temperament in 97 infants (Mayseless and Scher 2000). These studies provide some evidence that maternal separation anxiety is related to child separation anxiety (Dallaire and Weinraub 2005; Hock et al. 2004; Mayseless and Scher 2000; Peleg et al. 2006), but it remains unclear how these anxieties (Cartwright-Hatton et al. 2006)are transferred from mother to child.

One construct that may function as an explanatory mechanism of the link between maternal separation anxiety and child separation anxiety is psychologically controlling parenting. Psychological control is an intrusive parenting tactic, characterized by pressuring and manipulative strategies such as love withdrawal, guilt induction, and conditional approval (Barber 1996). There is robust evidence showing that psychological control is related to internalized distress in adolescents (Barber et al. 2005; Soenens et al. 2008), and some in children Stone et al. (2013b). In this study, we argue that one specific form of psychological control may explain the link between maternal and child separation anxiety. Subsequently, Soenens et al. (2010) proposed that maternal difficulties with interpersonal relatedness and closeness may lead to specific controlling parenting tactics, termed dependency oriented psychological control. It is argued that love and care are made contingent on the child’s dependence on the parents. Indeed, dependency-oriented psychological control was strongly related to parental anxiety regarding separation in adolescents (Soenens et al. 2010).

Regarding dependency-oriented psychological control and child separation anxiety, theory and research also suggest a link between these constructs. Psychological control is hypothesized to represent a threat to the child’s emerging sense of self (Barber 1996), as the child may be unable to develop a stable representation of the mother as a caring person. This unstable representation of the mother may lead to fears of loss of love and abandonment when the child attempts to separate from the parent (Blatt 2004), potentially leading to difficulties in distancing, interpersonal differentiation, and boundary-formation for the child (Hock and Schirtzinger 1992). In line with the notion that parenting tactics aimed at keeping the child in close proximity are associated with anxiety regarding separation, it was found that dependency-oriented psychological control was related to dependent personality features and depressive symptoms in adolescents (Soenens et al. 2010). Another proposed mechanism is that children of intrusive and controlling parents lack experience with independence and may perceive novel and ambiguous situations as threatening, which may provoke anxiety in separation-laden contexts (Wood 2006). Accordingly, it has been found that intrusive parenting and separation anxiety are related in children (Wood 2006), and adolescents (Kins et al. 2011; Mayseless and Scharf 2009).

In sum, we argued that maternal psychological control may be an intervening variable between maternal separation anxiety and her child’s separation anxiety. To our best knowledge, only one study explicitly tested this proposed mechanism. In this study, the relation between maternal separation anxiety and separation-individuation pathology in a large sample of emerging adults was found to be partially mediated by dependency oriented psychological control (Kins et al. 2011). However, no studies have tested this mechanism in childhood, when separation anxiety is most salient (Cartwright-Hatton et al. 2006). The present study sought to answer the following research questions. First, is maternal separation anxiety related to separation anxiety in children? Second, is this relationship mediated by dependency oriented psychological control? Third, are these relations moderated by gender? Based on previous studies, we expect that maternal separation anxiety is associated with child separation anxiety and partially mediated by dependency oriented psychological control. In accordance with the notion that cultural stereotypes render females more vulnerable to problems with interpersonal relatedness and dependency, we hypothesize that mothers use more dependency promoting techniques towards their daughters (Blatt 2004), and that associations between maternal separation anxiety and their daughters’ separation anxiety are stronger than between mothers and sons.

## Method

### Participants

In this study, 300 children were interviewed during the first measurement (T1). One child was excluded due to missing data and another child because she was over 8 years old. One year later (T2), 288 of these children (96 %) were re-interviewed, of whom one was excluded because of her advanced age. This resulted in a sample of 298 children at T1, and 287 children at T2. Of these participating children, 50 % was male and the mean age was 6.95 years (*SD* = 1.13; range 5–8 years). The majority of the children was of Dutch origin (97.4 %) and grew up in a two-parent family (92.2 %). Parents (T1 *n* = 289, T2 *n* = 269) completed questionnaires about the children at both time points. The parents who filled out the questionnaires were on average 38.29 years old (*SD* = 3.88), and 92.9 % of them were female. Over half of the parents were highly educated (54.8 %), 37.3 % had an intermediate education level, and 6.6 % lower education. Slightly over 1 % received some other type of education.

### Procedures

Longitudinal data [2011 (T1)–2012 (T2)] from the Child in Sight project were used (Stone et al. 2013a), which was approved by the committee on ethics from the Radboud University Nijmegen. Within this project, information was collected via children and their parents. Informed consent from the children’s parents was obtained. Each year, the Berkeley Puppet Interview (BPI; Measelle et al. 1998) was administered to the children by five certified master students or researchers. They all completed a training course in which the interviewing techniques of the BPI were extensively practiced. Subsequently, they each conducted eight practice interviews, and were then evaluated. The interviews were administered at primary schools in January and February of 2011 and 2012. Children were interviewed in an empty classroom to ensure confidentiality. Interviews were videotaped and after completion, the children were given a pair of stickers to thank them for their participation.

### Measures

#### Maternal Separation Anxiety

At both waves, mothers rated their feelings of separation anxiety concerning their child (MSAS; Hock et al. 1989). The subscale Maternal Separation Anxiety, which consists of 21 items, was used for this study. Each item is answered on a 5-point Likert scale, ranging from strongly disagree (1) to strongly agree (5). Sample items include ‘My child is happier when s/he is with me than when s/he is with the babysitter or teacher’ and ‘When I’m not with my child, I don’t have fun’. Psychometric properties of the MSAS have been found adequate. Cronbach’s alpha ranged from .83 to .84.

#### Separation Anxiety

The BPI is an interactive age-appropriate interviewing technique eliciting self-perceptions from 3.5 to 8 year-olds. The BPI has proven a reliable and valid instrument to assess child psychopathology and parenting (Measelle et al. 1998; Morris et al. 2002; Ringoot et al. 2013). BPI psychopathology scores are correlated to measures of parent- and teacher rated psychopathology (*r* .30–.40, respectively) (Measelle et al. 1998). During the actual BPI children are interviewed by using two identical dog hand puppets, named Iggy and Ziggy. Before the interview starts, the puppets introduce themselves and explain the interview in a playful way. By using three practice items the interviewer judges whether the procedure is clear to the child and then proceeds to the interview or repeats the practice items until the procedure is clear. An example of a practice item is: Puppet one: ‘I like chocolate’, Puppet two: ‘I don’t like chocolate. How about you?’. Throughout the interview the puppets thus make opposing statements about themselves and then ask the child ‘How about you?’. The puppet with whom the child agrees then repeats the child’s answer, thereby appraising the child’s answer.

Nuance was given to the BPI scores as interviews were coded by four trained observers on a seven-point scale. Responses that reflect the absence of separation anxiety or perceptions of dependency-oriented psychological control are coded, five, six, or seven, depending on the weight the child puts in its answer. Whereas a seven would reflect the highest end of absence (e.g., I never get scared if my mom or dad goes somewhere without me), the six would reflect the neutral absence response and the five a hesitant response (e.g., Most of the time, I don’t get scared if my mom or dad goes somewhere without me). At the opposite end of the spectrum, one, two, or three, reflect presence of psychopathology or presence of particular parent behaviors. Here one refers to the highest end of presence (e.g., I always get scared if my mom or dad goes somewhere without me), two reflects the neutral presence response and three again reflects a hesitant response indicating presence of psychopathology (e.g., Most of the time, I get scared if my mom or dad goes somewhere without me). When a child is not able to choose either one of the statements, this is coded a four. To test whether coders were reliable, 15 % of the videos were double-scored. Inter-rater agreement was satisfactory (ICC .83–.93). Separation anxiety in children was measured with six items. Example items are ‘When I’m at school I don’t miss my mom/When I’m at school I miss my mom’.

#### Dependency Oriented Psychological Control

Child perceptions of dependency oriented psychological control were measured using adapted versions of the ‘Psychological Control’ Scale-Youth Self-Report (PCS-YSR; Barber 1996), ‘Parental Regulation Scale’-Youth Self-Report (PRS-YSR; Barber 2002), and the Acceptance-Rejection scale from the Child Report of Parent Behaviors Scale (CRPBI; Schaefer 1965), respectively. A validated Dutch translation of each of these scales was available (Soenens et al. 2006). A panel of four people independently adapted the items to the children’s developmental level and to match the BPI format. Subsequently, the adapted items were discussed in an expert panel and the final items were selected through consensus. The parenting dimensions were scored as described above for the BPI items. Dependency-oriented psychological control was measured with four items. Example items are: ‘When I upset my mom she will talk to me like she normally does/When I upset my mom she will only talk to me when I’m nice to her’, ‘When I’m telling a story, my mom does not interrupt me/When I’m telling a story, my mom interrupts me’.

### Data Analyses

First, Pearson’s correlations were computed between all study variables. Second, three path models were estimated in Mplus version six (Muthén and Muthén 1998–2007) to evaluate the effects of maternal separation anxiety on children’s separation anxiety via dependency-oriented psychological control. The first model evaluated these associations cross-sectionally at T1. The second two models evaluated these associations longitudinally, with the second model not controlling for T1 separation anxiety in children, and the third model controlling for T1 separation anxiety in children. Model fit was assessed with various fit indices, including robust Chi-square with estimated degrees of freedom (df), comparative fit index (CFI; Bentler 1990), root mean squared error of approximation (RMSEA; Byrne 1998), and Tucker–Lewis index (TLI; Tucker and Lewis 1973). Direct associations between variables were assessed based on standardized path coefficients and *p* values. Indirect effects (i.e., mediation) were tested using a bootstrap method in Mplus (Shrout and Bolger 2002). Missing values on predictor variables were substituted in Mplus using full information maximum likelihood (FIML) estimation.

To examine gender differences in individual model paths, a freely estimated model was compared to a model in which parameters were constrained to be equal for boys and girls. If a significantly worse fit to the data was found for the constrained model, we employed a stepwise approach, such that each of the parameters were tested separately for sex differences. A Chi-square difference test was used to test relative model fit (Satorra and Bentler 2001).

## Results

Maternal separation anxiety at T1 was strongly related to maternal separation anxiety at T2 (Table 1). Further, dependency-oriented psychological control at T1 was related to dependency-oriented psychological control at T2. A similar association was found for separation anxiety, indicating temporal stability of these problems in young children. Maternal separation anxiety at was related to dependency-oriented psychological control and separation anxiety concurrently, but not longitudinally. Dependency-oriented psychological control was related to maternal separation anxiety and separation anxiety in children both concurrently and longitudinally. At both waves, more separation anxiety was reported by girls than boys, respectively [*t*(296) = −2.38, *p* < 0.05] and [*t*(285) = −2.82, *p* < 0.01]. No gender differences were found for the reports of maternal separation anxiety and dependency-oriented psychological control. Finally, on average, children reported more separation anxiety at T1 than at T2 [*t*(284) = 4.18, *p* < 0.01], and more maternal separation anxiety was reported at T1 than at T2 [t(217) = 3.15, *p* < 0.01].

**Table 1:** Correlations between all study variables

		*M (SD)*	1	2	3	4	5	6
1	Maternal separation anxiety T1	2.50 (.38)	–					
2	DPC T1	3.15 (.94)	.15*	–				
3	Separation anxiety T1	3.36 (.91)	.16*	.25**	–			
4	Maternal separation anxiety T2	2.43 (.38)	.77**	.12*	.17**	–		
5	DPC T2	3.10 (.90)	.09	.19**	.13*	.01	–	
6	Separation anxiety T2	3.07 (.98)	.10	.14*	.38**	.13*	.23**	–
7	Age T1	6.95 (1.13)	.03	.08	−.08*	−.04	−.06	−.15**

*DPC* dependency-oriented psychological control

** *p* < .01; * *p* < .05

The cross-sectional mediation model had adequate fit (χ^2^ (2) = 2.27, *p* = .32; *CFI* = .990; *RMSEA* = .023 (CI .000–.128); TLI = .966). A positive trend was found between maternal separation anxiety and separation anxiety in children (beta = .13, SE = .90, *p* = .05). Dependency-oriented psychological control was positively related to separation anxiety in children (beta = .24, SE = .09, *p* = .001). Maternal separation anxiety and dependency-oriented psychological control were positively marginally related (beta = .13, SE = .65, *p* = .059). Further, age was negatively related to separation anxiety in children (beta = −.13, SE = .27, *p* = .021), such that younger children reported more separation anxiety than older children. Gender was not related to separation anxiety (beta = .10, SE = .65, *p* = .10). The association between maternal separation anxiety and child separation anxiety was not statistically mediated by dependency-oriented psychological control (indirect effect = .03, SE = .02, *p* = .09). The model explained 10.4 % of the variance of separation anxiety in children.

In the first longitudinal model, we did not control for separation anxiety at T1. This model had adequate fit (χ^2^ (2) = 2.52, *p* = .324; *CFI* = .987; *RMSEA* = .022 (CI .000–.127); TLI = .956). Maternal separation anxiety at T1 was not related to separation anxiety in children at T2 (beta = .07, SE = .96, *p* = .24). Dependency-oriented psychological control at T1 was not related to separation anxiety in children at T2 (beta = .11, SE = .11, *p* = .09). Maternal separation anxiety at T1 was marginally related to dependency-oriented psychological control concurrently (beta = .13, SE = .64, *p* = .056). Further, age was again negatively related to separation anxiety at T2 (beta = −.19, SE = .31, *p* = .001), such that younger children reported more separation anxiety than older children. Gender was positively related to separation anxiety at T2 (beta = .17, SE = .74, *p* = .007), such that girls reported more separation anxiety than boys. The association between maternal separation anxiety at T1 and child separation anxiety at T2 was not statistically mediated by dependency-oriented psychological control (indirect effect = .02, SE = .01, *p* = .26). The model explained 7.7 % of the variance of separation anxiety at T2 in children.

The model in which we controlled for separation anxiety at T1 showed adequate fit to the data (χ^2^ (1) = .001, *p* = .97; *CFI* = 1.000; *RMSEA* = .000 (CI .000–.000); TLI = 1.122). Separation anxiety in children at T1 was associated with child separation anxiety at T2 (beta = .32, SE = .07, *p* = .000). Maternal separation anxiety at T1 was not related to separation anxiety in children at T2 (beta = .03, SE = .87, *p* = .57). Dependency-oriented psychological control at T1 was not related to separation anxiety in children at T2 (beta = .05, SE = .10, *p* = .44). Maternal separation anxiety at T1 was positively related to dependency-oriented psychological control concurrently (beta = 4.37, SE = 13.9, *p* = .003). Further, age was again negatively related to separation anxiety at T2 (beta = −.16, SE = .28, *p* = .003), and gender was positively related to separation anxiety at T2 (beta = .14, SE = .63, *p* = .011). The association between maternal separation anxiety at T1 and child separation anxiety at T2 was not statistically mediated by dependency-oriented psychological control (indirect effect = .22, SE = .17, *p* = .20). The model explained 18 % of the variance of separation anxiety at T2 in children.

Imposing constraints on the path loadings did not result in statistically significant model comparisons; the constrained models did not differ from the unconstrained models. These results indicate that the path coefficients do not differ among boys and girls.

## Discussion

The current study investigated whether maternal separation anxiety and separation anxiety in children were related cross-sectionally and longitudinally in a sample of five-nine-year-olds using innovative and age-appropriate measures for child-reports. Second, we investigated whether this relation was mediated by dependency-oriented psychological control. Results showed that maternal separation anxiety was related to separation anxiety in children cross-sectionally, albeit weakly, and not longitudinally. Moreover, dependency-oriented psychological control was related to separation anxiety in children cross-sectionally, but not longitudinally. Maternal separation anxiety was marginally related to dependency-oriented control. Further, there was no mediation of dependency-oriented psychological control, nor cross-sectionally or longitudinally. In conclusion, our findings do not support our hypotheses.

Studies on the association between maternal and child separation anxiety are scarce, and the available studies are hampered by small sample sizes (Dallaire and Weinraub 2005; Hock et al. 2004; Mayseless and Scher 2000; Peleg et al. 2006). Moreover, these studies were conducted in different developmental periods, making it hard to compare their findings. Although these studies were based on theoretical assertions (Blatt 2004; Hock et al. 1989), the theory is quite unspecific as to *when* maternal separation anxiety should impact children, and for *whom* this may be most disturbing. In the current study, we found that maternal separation anxiety was weakly related to separation anxiety in children, and we could not replicate previous findings regarding an association between maternal separation anxiety and separation anxiety in children over time. This shows that the available evidence for a link between maternal separation anxiety and separation in children is not supported. Although Dallaire and Weinraub (2005) found a quite strong association between maternal separation anxiety and child separation anxiety in children over time, in the same developmental period as the children in our study, these authors measured maternal separation anxiety during infancy. This may be important, as it may indicate that current feelings of maternal separation anxiety may be not as important as feelings of maternal separation anxiety earlier in development. This reasoning coincides with the theoretical proposition that the first process of separation-individuation is hypothesized to take place during infancy (Mahler 2000), and with attachment research, where ample studies have shown that early mother–child interactions shape children’s ability to regulate their emotions (e.g., Bakermans-Kranenburg et al. 2003).

Therefore, other factors may be more important predictors of separation anxiety at this age than maternal separation anxiety. Although it is unclear whether well-known parenting tactics, such as low responsiveness and behavioral control predict separation anxiety in children, maternal sensitivity has been reported as predicting separation anxiety (Dallaire and Weinraub 2005). Further, scholars using a cognitive framework for studying separation anxiety have shown that separation-related interpretive biases in children are related to childhood separation anxiety (Bögels et al. 2003; In-Albon et al. 2009, 2010; Perez-Olivas et al. 2011). However, these findings may be hard to replicate in more ecologically valid research, such as the current study. Also, these experimental studies did not test whether these biases predict separation anxiety in children over time.

Second, dependency-oriented psychological control did not mediate the relation between maternal separation anxiety and child separation anxiety. This contradicts the findings of Kins et al. (2011), who found dependency-oriented psychological control partially mediated the relation between maternal separation and separation issues in early adults. This divergence in findings may be due to the developmental period at hand. Our study focuses on early childhood where the child is not yet expected to separate from the mother. The study of Kins et al. focuses on early adults, where disconnecting from the parent and becoming independent is a salient developmental task (Arnett 2000). Possibly, younger children do not experience their mothers as being afraid of distancing, as these children *are* usually in close proximity to their mother.

Third, we did not find that children experienced their mothers to use more dependency-oriented psychological control toward girls than boys. Further, associations between maternal separation anxiety and separation anxiety in children were not stronger for girls than for boys. Thus, our hypothesis regarding the moderating role of gender in separation anxiety was not supported. Therefore, the notion that females may be more vulnerable to problems with interpersonal relatedness and dependency due to cultural stereotyping (Blatt 2004), was not confirmed by this study. As no previous studies tested this hypothesized association (Blatt 2004), these findings primarily call for replication of studies using a similar design and sample.

A number of limitations to this study should be noted. First, our measure of dependency-oriented psychological control is rather short. This may impede the reliability and validity of this scale. Thus, it is questionable whether we really measured what we intended to measure. This being said, correlations show that there is stability of this construct, and that it is correlated to measures it is theoretically expected to be associated with. Still, future research should study this construct in adolescents, such that a well-validated self-report instrument can be employed (e.g. Soenens et al. 2010). Second, although we used multiple informants in this study, we did not include alternative measures of both our maternal and separation anxiety measures, and dependency-oriented psychological control. It has been argued that when measuring a construct, multiple methods should always be employed, as to be certain of the veracity of the measurement (De Los Reyes 2013). Such alternative methods could include questionnaires, but also observations of parent–child interactions (Barber 1996). Third, our sample is biased in that roughly half of our participants are highly educated. This may have led to typical problems found with highly educated samples, namely that there is less variance in problem behaviors.

In conclusion, this study found no associations between maternal separation anxiety and separation anxiety in children over time in young children, while using a large sample and different informants. Also, we did not find support for the mediating role of dependency-oriented psychological control. Future research is warranted in order to draw firm conclusions about the relations between maternal and child separation anxiety, and the possible mediating role of dependency-oriented psychological control. For future research the recommendations mentioned above should be taking into account.
